# Determination of Polycyclic Aromatic Hydrocarbons in Industrial Harbor Sediments by GC-MS

**DOI:** 10.3390/ijerph9062175

**Published:** 2012-06-11

**Authors:** Cheng-Di Dong, Chih-Feng Chen, Chiu-Wen Chen

**Affiliations:** Department of Marine Environmental Engineering, National Kaohsiung Marine University, 142 Haijhuan Road, Nanzih District, Kaohsiung City 81157, Taiwan; Email: cddong@mail.nkmu.edu.tw (C.-D.D.); dong3762@mail.nkmu.edu.tw (C.-F.C.)

**Keywords:** PAHs, GC-MS, harbor sediment

## Abstract

Analysis of the 16 polycyclic aromatic hydrocarbons (PAHs) of the US Environmental Protection Agency priority pollutant list was carried out in sediment samples of an industrial port in the southern Kaohsiung Harbor of Taiwan which is supposed to be extensively polluted by industrial wastewater discharges. The determination and quantification of PAHs in sediment samples were performed using gas chromatography coupled to mass spectrometry (GC-MS) with the aid of deuterated PAH internal standards and surrogate standards. The total concentrations of the 16 PAHs varied from 4,425 to 51,261 ng/g dw, with a mean concentration of 13,196 ng/g dw. The PAHs concentration is relatively high in the river mouth region, and gradually diminishes toward the harbor region. Diagnostic ratios showed that the possible source of PAHs in the industrial port area could be coal combustion. As compared with the US Sediment Quality Guidelines (SQGs), the various observed levels of PAHs exceeded the effects range median (ERM), and could thus cause acute biological damages. The results can be used for regular monitoring, and future pollution prevention and management should target the various industries in this region for reducing pollution.

## 1. Introduction

Polycyclic aromatic hydrocarbons (PAHs) are included in the European Union and US Environmental Protection Agency priority pollutant lists because PAHs represent the largest group of compounds that are mutagenic, carcinogenic, and teratogenic [1,2]. They could also pose potential threats to the marine environment. The effect of PAHs is usually widespread and permanent in environmental media. Most PAHs have high hydrophobicity, and can be sorbed strongly by water-borne organic and inorganic particles. They may eventually be brought down to the bottom sediment as a sink in the aquatic system; the PAHs found in the sediments are resistant to bacterial degradation in an anoxic environment. Even under favorable conditions, the sorbed PAHs will be released to the water as an extended source to threaten the aquatic ecosystem through bioaccumulation in food chains [3]. Thus, understanding the distribution, composition, and potential biological impacts is essential and important for appropriately managing PAHs levels in the environment.

**Figure 1:**
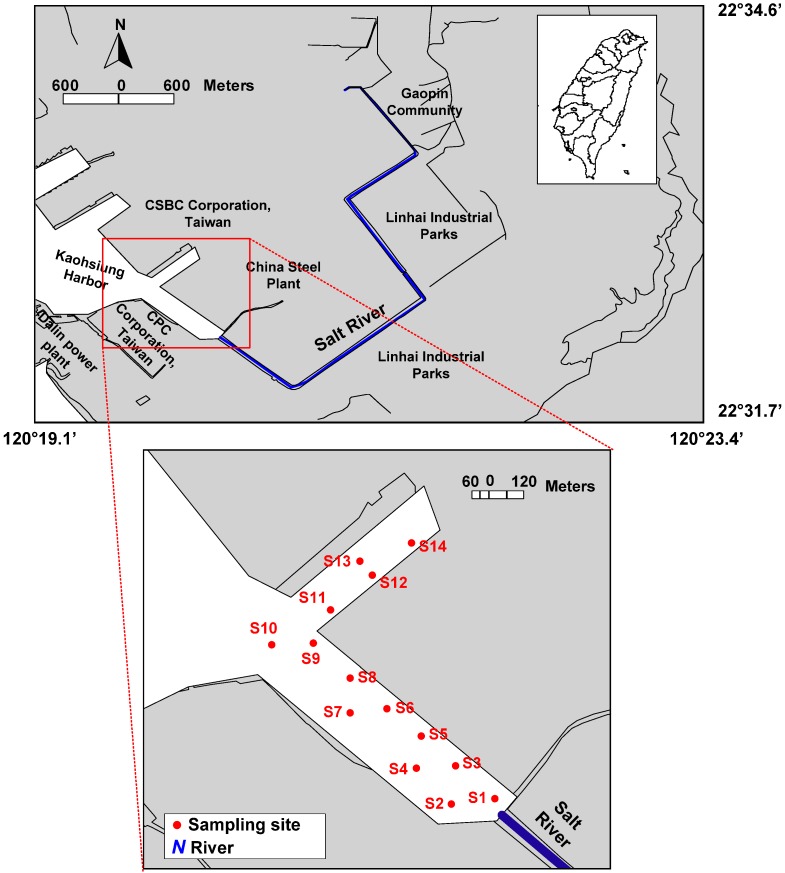
Map of the study area and sampling locations.

Kaohsiung Harbor is situated along the southwestern coast, and it is the largest international port in Taiwan. In addition, it receives effluents from four contaminated rivers, including Love River, Canon River, Jen-Gen River, and Salt River. Among these four rivers, the Salt River flows through the Linhai Industrial Park and the China Steel Plant (the largest steel plant in Taiwan) and is finally discharged into southern Kaohsiung Harbor (Figure 1). In the Linhai Industrial Park, there are more than 482 registered industrial factories that discharge their treated and untreated wastewaters into the Salt River. Results from recent investigations indicate that the industrial port area of southern Kaohsiung Harbor is heavily polluted by PAHs, and the upstream pollutants brought over by the Salt River represent one of the major pollution sources [4,5]. The river receives untreated municipal and industrial wastewater discharges causing serious deterioration of the river water quality and the environmental quality near the river mouth that seriously threaten the water environmental ecological system.

Previously research on PAHs contamination in the surface sediments of Kaohsiung Harbor reported that the highest levels of PAHs were recorded for surface sediment samples collected in the vicinity of river mouth situated in industrial port area of southern Kaohsiung Harbor, indicating more PAHs were accumulated in industrial port area sediments [4,5]. However, the PAH contamination had significant spatial and temporal variations in harbor sediments, and more understanding of the contamination is needed [6]. The present study therefore aimed to investigate: (a) the distribution, composition, and relative pollution levels of PAHs in the sediments of industrial port area in the southern Kaohsiung Harbor, (b) identify possible sources of PAHs and (c) evaluate the potential biological impacts of these pollutants on the environment.

## 2. Materials and Methods

### 2.1. Sampling

Surface sediment samples were collected at 14 stations located at the industrial port area of southern Kaohsiung Harbor in January 2007 (Figure 1) with an Ekman Dredge Grab aboard a fishing boat. Immediately after collection, the samples were scooped into glass bottles, which had been pre-washed with *n*-hexane and kept in an icebox, and then transported to the laboratory for analysis. In the laboratory, the samples were freeze-dried for 72 h, ground to pass through a 0.5 mm sieve and fully homogenized [4,5]. The dried sediments were placed at -20 °C in amber glass bottles pre-washed with *n*-hexane and covered with solvent-rinsed aluminum foil until further processing and analysis.

### 2.2. Chemicals

All solvents and reagents used were of trace analysis (TA), chromatographic (HPLC) or ACS grade. Standards of 16 PAHs including naphthalene (NA), acenaphthylene (ACY), acenaphthene (ACE), fluorene (FL), phenantrene (PH), anthracene (AN), fluoranthene (FLU), pyrene (PY), benzo[a]anthracene (BaA), chrysene (CH), benzo[b]fluoranthene (BbF), benzo[k]fluoranthene (BkF), benzo[a]pyrene (BaP), indeno[1,2,3-cd]pyrene (IP), dibenzo[a,h]anthracene (DA), and benzo[g,h,i]perylene (BP) in a 80 mg/L mixture solution were obtained from AccuStandard Chem. Co. (New Haven, CT, USA). Deuterated PAH internal standard solutions (naphthalene-d_8_, acenaphthene-d_10_, phenanthrene-d_10_, chrysene-d_12_, and perylene-d_12_) at 4,000 mg/L and surrogate standard solutions (2-fluorobiphenyl and 4-terphenyl-d_14_) at 2,000 mg/L were obtained from AccuStandard Chem. Co. Internal and surrogate standards were used for sample quantification and quantifying procedural recovery.

PAHs working standards, internal standard mixture solutions and surrogate standard mixture solutions were properly diluted with HPLC grade *n*-hexane and prepared daily before the analysis. Glassware was washed before use with *n*-hexane and dried in an oven at 105 °C. Other materials were previously washed with ultrapure water and acetone.

### 2.3. Sample Preparation

Sediment samples were extracted using a procedure from Chen and Chen [4], which was slightly modified. Briefly, one g (accuracy ± 0.0001 g) of dry and homogenized sediment sample was put into a clean centrifuge tube, and a 1:1 (v/v) acetone/*n*-hexane (5 mL), and surrogate standard mixture (2-fluorobiphenyl and 4-terphenyl-d_14_) solutions were then added. Blanks were prepared following the same procedure without adding sediment sample. The standard sample used for quality control was prepared by adding the standard solution to 1:1 (v/v) acetone/*n*-hexane. All samples were vortexed for 1 min and the mixture was subject to ultrasonic treatment for 15 min for PAH extraction. The sample tubes were then centrifuged at 2,000 rpm for 10 min. After centrifuging, the organic layer containing the extracted compounds was siphoned out with a Pasteur pipette, and the sediment was re-extracted twice with 1:1 (v/v) acetone/*n*-hexane (5 mL). All extracts were pooled together, and activated copper was added to the combined extract for desulphurization. After subsequent drying over anhydrous sodium sulphate, and concentration to 1.0 mL using a gentle stream of nitrogen, an internal standard mixture (naphthalene-d_8_, acenaphthene-d_10_, phenanthrene-d_10_, chrysene-d_12_, and perylene-d_12_) solution was added to the extract to be analyzed using gas chromatography with mass selective detection (GC-MS). Between this study and the previous work [4] the main difference was that in our case the internal standards were increased from three to five types and the capillary column and GC analysis conditions were different too. Moreover, concentrations of PAHs were corrected for the surrogate standard recoveries in this study.

### 2.4. GC-MS Instrumentation and Conditions

An Agilent 6890N GC (Agilent Technologies, Santa Clara, CA, USA) equipped with an Agilent 7683B Injector (Agilent Technologies, Santa Clara, CA, USA), a 30 m, 0.25 mm i.d. HP-5MS capillary column (Hewlett-Packard, Palo Alto, CA, USA) coated with 5% phenyl-methylsiloxane (film thickness 0.25 μm) and an Agilent 5975 mass selective detector (MSD) was used to separate and quantify the PAH compounds. The samples were injected in the splitless mode at an injection temperature of 300 °C. The transfer line and ion source temperatures were 280 °C and 200 °C. The column temperature was initially held at 40 °C for 1 min, raised to 120 °C at the rate of 25 °C/min, then to 160 °C at the rate of 10 °C/min, and finally to 300 °C at the rate of 5 °C/min, held at final temperature for 15 min. Detector temperature was kept at 280 °C. Helium was used as a carrier gas at a constant flow rate of 1 mL/min. Mass spectrometry was acquired using the electron ionization (EI) and selective ion monitoring (SIM) modes. The ion mass program used for quantification is detailed in Table 1.

**Table 1:** GC-MS conditions under time scheduled selected ion monitoring.

Time window (min)	Compound	No. of rings	Retention time (min)	Molecular mass	Ions	m/z window
4.00–9.45	Naphthalene-d_8_ (IS1)	2	7.021	136	136	127,128,129,136,172
	Naphthalene	2	7.052	128	128, 129,127	
	2-Fluorobiphenyl (SS1)	2	8.297	172	172	
9.45–13.50	Acenaphthylene	3	10.128	152	152, 151,153	151,152,153,154,164,166,167
	Acenaphthene-d_10_ (IS2)	3	10.495	164	164	
	Acenaphthene	3	10.577	154	154, 153,152	
	Fluorene	3	12.049	166	166, 165,167	
13.50–21.50	Phenanthrene-d_10_ (IS3)	3	15.250	188	188	101,176,178,179,188,200,202,203
	Phenanthrene	3	15.334	178	178, 179,176	
	Anthracene	3	15.526	178	178, 176,179	
	Fluoranthene	4	20.224	202	202, 101,203	
	Pyrene	4	21.164	202	202, 200,203	
21.50–29.00	4-Terphenyl-d_14_ (SS2)	4	22.179	244	244	226,228,229,240,244
	Benzo[a]anthracene	4	26.660	228	228, 229,226	
	Chrysene-d_12_ (IS4)	4	26.699	240	240	
	Chrysene	4	26.813	228	228, 226,229	
29.00–51.20	Benzo[b]fluoranthene	5	31.321	252	252, 253,125	125,138,139,252,253,276,277
	Benzo[k]fluoranthene	5	31.431	252	252, 253,125	
	Benzo[a]pyrene	5	32.587	252	252, 253,125	
	Perylene-d_12_ (IS5)	5	32.827	264	264	
	Indeno[1,2,3-cd]pyrene	6	36.683	276	276, 138,277	
	Dibenz[a,h]anthracene	5	36.820	278	278, 139,279	
	Benzo[g,h,i]perylene	6	37.616	276	276, 138,277	

### 2.5. Identification and Quantification

Identity of PAHs in the samples was confirmed by the retention time and abundance of quantification/confirmation ions in the authentic PAHs standards. Sixteen priority PAHs were quantified using the response factors related to the respective internal standards based on five-point calibration curve for individual compounds. In this study, the concentrations of PAHs were corrected for the surrogate standard recoveries, and are expressed on a dry-weight (dw) basis.

## 3. Results and Discussion

### 3.1. Analytical Characteristics

Five-point calibration curve (0.08 to 4 ng), procedural blank, check standard and sample duplicates were carried out for every set of samples. The response factors based on the five-point calibration curve for individual compounds showed acceptable relative standard deviation (RSD) values (1.1 to 14.1%), the procedural blank values were always less than the detection limit, the recoveries of individual PAHs in check standards ranged from 87 ± 6% to 128 ± 4% (n = 3) and the relative percent differences of sample duplicates ranged from 7.0 ± 6.0% to 13.3 ± 3.6% (n = 3) for all of the target analyses (Table 2). The surrogate standard recoveries were 94.1 ± 6.6% for 2-fluorobiphenyl and 108.4 ± 8.2% for 4-terphenyl-d_14_ with sediment samples (n = 17). The detection limits of the analytical procedure were estimated from three times standard deviation from repeated (n = 7) analysis of 16 PAHs (8 pg), and the amount of sample extracted. The detection limits were 0.6 (FL)–5.4 (DA) ng/g dry weight for individual PAHs (Table 2). Reference materials SES-1 (polycyclic aromatic hydrocarbons in spiked estuarine sediment) from National Research Council of Canada (NRCC) were used. Certified and measured concentrations are showed in Table 3 and there is a good agreement among results being the error below 20% for individual PAHs.

**Table 2:** Response factor, detection limits, recoveries of check standards, and relative percent differences of sample duplicates for individual PAHs in this study.

Compound	Response factor (RF) (n = 5)		Detection Limits DL (ng/g)	Check analysis (n = 7) R ^a^ (%)	Duplication analysis (n = 7) RPD ^a^ (%)
	Average ± SD ^a^	RSD ^a^ (%)			
Naphthalene	2.08 ± 0.17	8.4	2.9	122 ± 12	8.7 ± 4.6
Acenaphthylene	1.85 ± 0.11	5.9	1.4	87 ± 6	11.8 ± 3.8
Acenaphthene	1.14 ± 0.05	4.4	1.9	107 ± 9	9.2 ± 5.0
Fluorene	0.86 ± 0.01	1.1	0.6	98 ± 3	11.2 ± 5.4
Phenanthrene	1.09 ± 0.14	12.9	2.3	105 ± 9	10.7 ± 3.3
Anthracene	1.28 ± 0.10	7.6	2.0	89 ± 8	7.0 ± 6.0
Fluoranthene	1.17 ± 0.06	4.8	1.8	90 ± 9	8.9 ± 2.4
Pyrene	1.22 ± 0.07	5.9	1.7	90 ± 8	12.3 ± 2.3
Benzo[a]anthracene	0.97 ± 0.11	11.6	2.2	105 ± 9	13.3 ± 3.6
Chrysene	1.47 ± 0.20	13.7	2.2	105 ± 8	11.9 ± 7.2
Benzo[b]fluoranthene	0.79 ± 0.09	11.9	3.5	120 ± 16	12.3 ± 4.3
Benzo[k]fluoranthene	1.39 ± 0.14	10.2	3.0	107 ± 14	10.8 ± 2.1
Benzo[a]pyrene	0.78 ± 0.03	4.2	3.5	91 ± 16	12.3 ± 4.6
Indeno[1,2,3-cd]pyrene	0.64 ± 0.06	9.3	4.4	103 ± 18	12.4 ± 6.4
Dibenz[a,h]anthracene	0.41 ± 0.06	14.1	5.4	112 ± 14	11.2 ± 4.9
Benzo[g,h,i]perylene	0.72 ± 0.07	9.9	5.3	128 ± 4	10.1 ± 7.4
2-Fluorobiphenyl (SS1)	1.52 ± 0.09	5.86	-	102 ± 7	7.5 ± 2.5
4-Terphenyl-d_14_ (SS2)	1.41 ± 0.10	7.09	-	107 ± 18	9.2 ± 4.0

^a^ SD: standard deviation; RSD: Relative standard deviation; R: Recoveries; RPD: Relative percent differences.

**Table 3:** Errors of individual PAHs in reference materials SES-1 (polycyclic aromatic hydrocarbons in spiked estuarine sediment) made in this study.

Compounds	Measured concentration (ng/g dw)			Certified equate concentration (ng/g dw)	Error ^a^ (%)
	#1	#2	#3		
Naphthalene	1,882	1,923	1,905	1,700	12.0 ± 1.2
Acenaphthene	624	688	645	590	10.6 ± 5.5
Fluorene	631	623	615	550	13.3 ± 1.5
Phenanthrene	1,121	1,165	1,103	1,050	7.6 ± 3.0
Anthracene	15	17	16	20	20.0 ± 5.0
Fluoranthene	1,553	1,545	1,614	1,350	16.3 ± 2.8
Pyrene	2,311	2,382	2,154	2,400	4.9 ± 4.9
Benzo[a]anthracene	425	410	425	500	16.0 ± 1.7
Chrysene	1,055	1,284	1,148	1,100	8.4 ± 7.2
Benzo[a]pyrene	176	165	133	150	12.9 ± 3.9
Benzo[g,h,i]perylene	592	619	612	690	11.9 ± 2.0
Dibenz[a,h]anthracene	541	537	587	600	7.5 ± 4.6
Indeno[1,2,3-cd]pyrene	727	766	773	800	5.6 ± 3.1

^a ^average ± standard deviation.

### 3.2. GC-MS Separation and Identification

**Figure 2:**
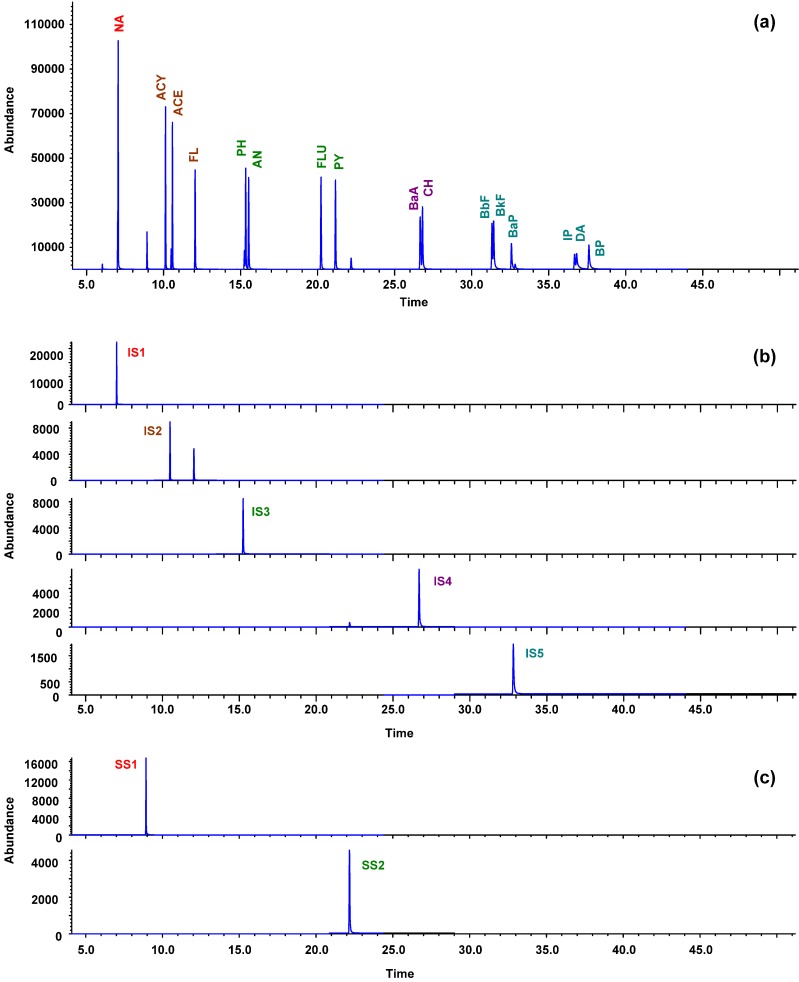
(**a**) GC-MS total ion chromatogram of sixteen PAHs, (**b**) selected ion chromatograms of the five internal standards, namely naphthalene-d_8_ (IS1), acenaphthene-d_10_ (IS2), phenanthrene-d_10_ (IS3), chrysene-d_12_ (IS4), and perylene-d_12_ (IS5), (**c**) two surrogate standards, 2-fluorobiphenyl (SS1) and 4-terphenyl-d_14_ (SS2).

Table 2 shows the experimental mass conditions used in the GC-MS analysis. Prior to analyzing the samples, the efficiency of GC-MS for analysis of the target compounds was tested with a standard mixture of 16 PAHs. Figure 2a shows the total ion chromatogram for this analysis. The identities of 16 PAHs were confirmed by the retention time and abundance of quantification/confirmation ions in the authentic PAHs standards. Since the 16 PAHs have significantly different chemical properties and retention times, five isotopic internal standards were used to monitor the 16 PAHs. Naphthalene-d_8_ with a retention time of 7.021 min was used for the NA. Acenaphthene-d_10_ with a retention time of 10.495 min was used for the ACY, ACE, and FL within the retention time window of 9.45–13.50 min. Phenanthrene-d_10_ with a retention time of 15.250 min was used for the PAHs within the retention time range of 13.50–21.50 min. Chrysene-d_12_ was used for CH and BaA. Perylene-d_12_ was used for the remaining PAHs. Figure 2(b,c) show the selected ion chromatograms illustrating how the internal standards and surrogate standards effectively cover the 16 PAHs. The separation and quantitation of PAHs in the sediment samples were achieved using the same GC-MS conditions as the standards. Sixteen PAHs were quantified using the response factors related to the respective internal standards based on five-point calibration curve for individual compounds.

**Table 4:** PAH concentration (ng/g dw)in surfaces sediments of industrial port area of southern Kaohsiung Harbor.

Station	2-ring	3-ring					4-ring				5-ring				6-ring		ΣLPAHs ^a^	ΣHPAHs ^a^	ΣPAHs ^a^
	NA	ACE	AC	FL	PH	AN	FLU	PY	BaA	CH	BbF	BkF	BaP	DA	IP	BP			
S1	1,211	292	1,412	1,370	3,181	206	2,475	1,795	1,925	1,273	1,688	965	957	37	186	354	7,671	11,654	19,325
S2	2,811	494	6,226	2,964	6,507	2,919	7,817	6,429	2,666	964	3,891	2,196	2,327	332	1,437	1,281	21,921	29,339	51,261
S3	1,916	396	4,262	2,869	5,256	2,593	6,036	4,950	3,522	2,311	2,638	1,489	1,492	240	925	768	17,291	24,370	41,661
S4	1,156	159	957	644	2,199	134	1,636	1,284	2,101	1,472	2,245	1,277	729	75	603	269	5,250	11,692	16,941
S5	915	5	888	792	2,630	154	2,035	1,550	1,344	1,245	1,495	1,102	539	22	197	220	5,384	9,749	15,133
S6	826	14	971	621	1,831	1,684	987	770	1,545	850	1,667	941	473	48	460	379	5,947	8,118	14,065
S7	2,360	296	2,469	1,420	3,733	2,329	6,468	4,552	1,999	2,154	3,386	1,910	217	189	320	324	12,608	21,519	34,127
S8	1,836	3	468	859	1,827	360	2,224	2,405	2,913	1,866	3,068	1,022	931	198	294	366	5,353	15,288	20,641
S9	973	8	751	723	2,223	2,092	1,447	1,189	1,359	902	1,660	936	1,035	54	208	482	6,771	9,272	16,043
S10	934	269	316	274	1,390	1,442	1,585	1,465	1,046	722	2,065	1,165	409	151	540	486	4,624	9,634	14,258
S11	440	52	376	184	557	205	460	381	274	149	345	362	154	177	143	167	1,814	2,611	4,425
S12	483	106	315	366	683	550	1,068	909	1,178	777	944	533	1,760	269	512	335	2,503	8,286	10,789
S13	873	4	598	344	1,245	90	836	663	470	324	1,100	621	314	40	292	289	3,155	4,948	8,103
S14	1,506	394	2,680	1,876	4,273	262	2,170	1,754	2,908	2,048	2,660	1,501	1,548	114	710	216	10,991	15,628	26,619
																			
ERL ^b^	160	44	16	19	240	85.3	600	665	261	384	–	–	430	63.4	–	–	552	1,700	4,022
ERM ^b^	2,100	640	500	540	1,500	1,100	5,100	2,600	1,600	2,800	–	–	1,600	260	–	–	3,160	9600	44,792

^a ^ΣLPAHs: sum of NA, ACE, AC, FL, PH, and AN; ΣHPAHs: sum of FLU, PY, BaA, CH, BbF, BkF, BaP, IP, DA, and BP; ΣPAHs: sum of 16 PAHs; ^b^ ERL and ERM refers to the effects range low and median [7].

### 3.3. Distribution and Composition of PAHs

**Figure 3:**
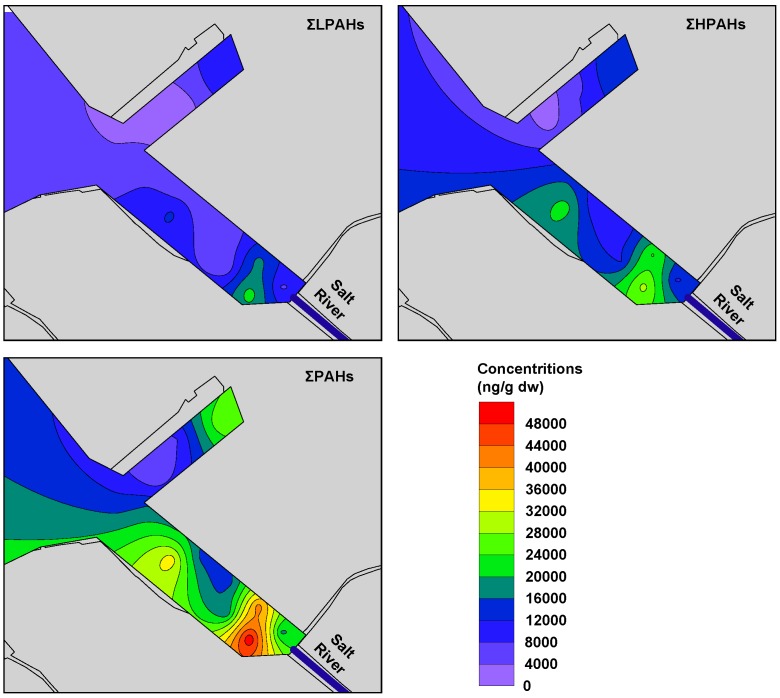
Distributions of ΣLPAH, ΣHPAH, andΣPAHsin sediments fromthe industrial port area of southern Kaohsiung Harbor.

The distribution of 16 PAHs in sediments of industrial port area of southern Kaohsiung Harbor is shown in Table 4. The total amount of PAHs (ΣPAHs) varied from 4,425 to 51,261 ng/g dw, with a mean concentration of 20,957±13,196 ng/g dw. In this study, the average ΣPAHs concentrations were higher than our previous work sampling in the same area in 2006 [4] and 2009 [5], when average ΣPAHs were 13,980 ± 3,254 ng/g dw (n = 3) and 14,616 ± 10,663 ng/g dw (n = 9), respectively. Concentration distributions of ΣPAHs in industrial port area sediment shown in Figure 3 reveal that the sediment PAHs content is relatively higher near the Salt River mouth, and gradually decreases in the direction toward the harbor. This indicates that the major sources of sediment PAHs came from the polluted urban rivers.

**Figure 4:**
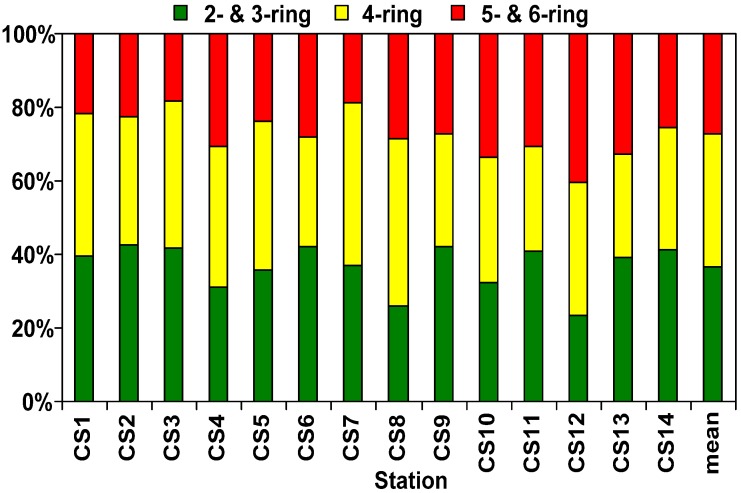
PAHs composition in sediments of industrial port area of southern Kaohsiung Harbor, 2- & 3-ring: NA, ACY, ACE, FL, PH, AN. 4-ring: FLU, PY, BaA, CH. 5- & 6-ring: BbF, BkF, BaP, IP, DA, BP.

According to the number of aromatic rings, the 16 PAHs were divided into three groups: (a) 2- & 3-ring, (b) 4-ring, and (c) 5- & 6-ring PAHs. The 2- & 3-ring PAHs were predominant in sediments from industrial port area of southern Kaohsiung Harbor, ranging from 23% to 43%, with mean of 37% (Figure 4); the percentage compositions are 28–48% (mean of 36%) and 18–40% (mean of 27% )for the 4-ring and 5- & 6-ring PAHs, respectively. The predominance of low and medium molecular weight PAHs in the sediments of this study area reflects the presence of significant combustion products from low temperature pyrolytic processes and/or petrogenic sources [5,8]. The PAHs pollutant level classification was suggested by Baumard *et al.* [9]: (a) low, 0–100 ng/g; (b) moderate, 100–1,000 ng/g; (c) high, 1,000–5,000 ng/g; and (d) very high, >5,000 ng/g. Sediments from this study area can be characterized as having high to very high PAH pollution. In this study, the composition of PAH congeners and pollution levels were similar to our previous works [4,5]. The result of the study can be confirmed that PAHs had both high extent pollutions and pollutant types in this area, and provided more accurate information for reference of the remediation strategies in the future.

**Figure 5:**
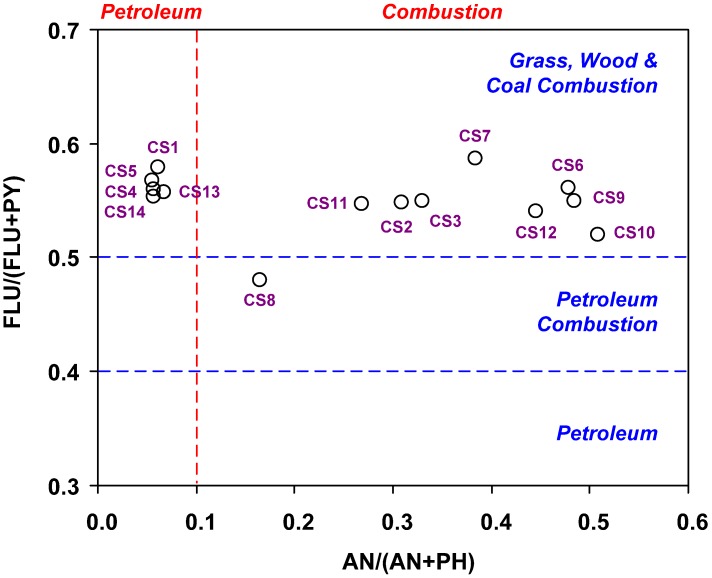
PAHs cross plots for the ratios of FLU/(PY + FLU) vs. AN/(AN + PH).

### 3.4. Sources of PAHs in Sediment

Several PAHs isomeric ratios have been used to identify different sources that contribute PAHs to environmental samples [4,5,10,11]. The common ratios used include AN/(PH + AN) [2,4,12,13,14], and FLU/(FLU + PY) [4,13,14,15]. Ratios of AN/(PH + AN) < 0.1 and FLU/(FLU + PY) < 0.4 usually imply a petrogenic source, whereas ratios of AN/(PH + AN) > 0.1 and FLU/(FLU + PY) > 0.5 suggest a pyrogenic source and combustion source of biomass (grass, wood, or coal combustion), respectively. If the FLU/(FLU + PY) ratio is between 0.4 and 0.5, a combustion of petroleum origin is suggested. 

Figure 5 shows the distribution AN/(PH + AN) and FLU/(FLU + PY) ratios in all sediment samples. Results show that ratios of AN/(PH + AN) and FLU/(FLU + PY) were <0.1 and >0.5, respectively at Stations S1, S4, S5, S13, and S14, suggesting that mixed sources could be possible source of PAHs; ratios of AN/(PH + AN) and FLU/(FLU + PY) at Stations 8 were higher than 0.1 and between 0.4 and 0.5, respectively indicate that petroleum combustion sources could be possible source of PAHs; ratios of AN/(PH + AN) and FLU/(FLU + PY) at other stations were higher than 0.1 and 0.5, respectively indicate that coal combustion would make the possible contributions to PAHs. Results from the ratio calculations suggest that PAH input to the industrial port area of southern Kaohsiung Harbor mainly came from domestic oil/coal combustion, because oil/coal burning was used for the energy source in this area [4]. Our previous works showed that coal combustion was the main source of PAHs in the study areas [4,5]. However, the oil combustion and some petrogenic characteristics were also found in the sediments that may be due to the more completed station used in the present study.

### 3.5. Sediment Biological Effects Based on PAHs

The widely used sediment toxicity screening guidelines of the US National Oceanic and Atmospheric Administration provide two target values to estimate potential biological effects: effects range low (ERL) and effect range median (ERM) [7]. The guideline was developed by comparing various sediment toxicity responses of marine organisms or communities with observed PAH concentrations in sediments. These two values delineate three concentration ranges for each particular chemical. When the concentration is below the ERL, it indicates that biological effects should be rare. If the concentration equals to or is greater than the ERL, but below the ERM, it indicates that a biological effect would occur occasionally. Concentrations at or above the ERM indicate that a negative biological effect would occur frequently. Table 4 shows the measured concentrations of PAHs in comparison with the ERM and ERL values. Among the 14 sediment samples collected, the ΣLPAHs is between ERL and ERM in 3 samples (21%), and exceed ERM in the other samples (79%); the ΣHPAHs is between ERL and ERM in five samples (36%), and exceed ERM in the other samples (64%); the ΣPAHs is between ERL and ERM in 13 samples (93%), and one sample (station S2) exceed ERM. For an individual PAH, they were above ERL but below ERM in three to 14 samples, which indicate that biological effects would occur occasionally. Moreover, except for Station 10 and 11, at least one type of PAHs exceeded the ERM in all stations, which indicates that biological effects would occur frequently.

In addition, a sediment quality guideline of 1,000 ng/g dw total PAHs to protect estuarine fish against several important health effects was suggested by Johnson *et al.* [16]. According to this guideline, the results of the present study show ΣPAHs exceed 1,000 ng/g dw at all sampling locations and management to reduce adverse environmental effects is urgent.

## 4. Conclusions

Analysis for 16 PAHs was carried out in sediment samples of an industrial port in the southern Kaohsiung Harbor (Taiwan). The distributions, possible sources and potential biological effects were also evaluated. The total concentrations of 16 PAHs varied from 4,425 to 51,261 ng/g dw, with a mean concentration of 13,196 ng/g dw. The PAH concentration is relatively high in the river mouth region, and gradually diminishes toward the harbor region. This indicates that the major sources of sediment PAHs came from the polluted urban rivers. The possible source of PAHs in the industrial port area could be coal combustion. As compared with the US Sediment Quality Guidelines (SQGs), several of the observed PAH levels exceeded the ERM, and could thus cause acute biological damage. The results should be useful in designing future strategies for environmental protection of the port, with special focus on the area at industrial zone dock. 
